# Assessing the effect of insecticide-treated cattle on tsetse abundance and trypanosome transmission at the wildlife-livestock interface in Serengeti, Tanzania

**DOI:** 10.1371/journal.pntd.0008288

**Published:** 2020-08-25

**Authors:** Jennifer S. Lord, Rachel S. Lea, Fiona K. Allan, Mechtilda Byamungu, David R. Hall, Jessica Lingley, Furaha Mramba, Edith Paxton, Glyn A. Vale, John W. Hargrove, Liam J. Morrison, Stephen J. Torr, Harriet K. Auty

**Affiliations:** 1 Dept. of Vector Biology, Liverpool School of Tropical Medicine, Liverpool, United Kingdom; 2 Roslin Institute, Royal (Dick) School of Veterinary Studies, University of Edinburgh, Edinburgh, United Kingdom; 3 Vector and Vector-Borne Diseases Research Institute, Tanga, Tanzania; 4 Natural Resources Institute, University of Greenwich, Chatham, United Kingdom; 5 SACEMA, University of Stellenbosch, Stellenbosch, South Africa; 6 Epidemiology Research Unit, SRUC, An Lochran, Inverness, IV2 5NA, United Kingdom; 7 Institute of Biodiversity, Animal Health and Comparative Medicine, University of Glasgow, Glasgow, United Kingdom; Kenya Agricultural and Livestock Research Organization, KENYA

## Abstract

In the absence of national control programmes against Rhodesian human African trypanosomiasis, farmer-led treatment of cattle with pyrethroid-based insecticides may be an effective strategy for foci at the edges of wildlife areas, but there is limited evidence to support this. We combined data on insecticide use by farmers, tsetse abundance and trypanosome prevalence, with mathematical models, to quantify the likely impact of insecticide-treated cattle. Sixteen percent of farmers reported treating cattle with a pyrethroid, and chemical analysis indicated 18% of individual cattle had been treated, in the previous week. Treatment of cattle was estimated to increase daily mortality of tsetse by 5–14%. Trypanosome prevalence in tsetse, predominantly from wildlife areas, was 1.25% for *T*. *brucei s*.*l*. and 0.03% for *T*. *b*. *rhodesiense*. For 750 cattle sampled from 48 herds, 2.3% were PCR positive for *T*. *brucei s*.*l*. and none for *T*. *b*. *rhodesiense*. Using mathematical models, we estimated there was 8–29% increase in mortality of tsetse in farming areas and this increase can explain the relatively low prevalence of *T*. *brucei s*.*l*. in cattle. Farmer-led treatment of cattle with pyrethroids is likely, in part, to be limiting the spill-over of human-infective trypanosomes from wildlife areas.

## Author summary

The acute form of sleeping sickness in Africa is caused by the parasite *Trypanosoma brucei rhodesiense*. It is transmitted by tsetse flies and can be maintained in cycles involving both livestock and wildlife as hosts. Humans are incidentally infected and are particularly at risk of infection near protected areas where there is both wildlife and suitable habitat for tsetse. In these regions, the tsetse vector cannot be eradicated, nor can infection be prevented in wildlife. Here we use field studies of tsetse and livestock in combination with mathematical models of tsetse population change and trypanosome transmission to show that use of pyrethroid-based insecticides on cattle–by farmers at the edge of protected areas–could be contributing to lowering the risk of sleeping sickness in Serengeti District, Tanzania. To our knowledge, our study is the first to report farmer-led tsetse control, co-incident with tsetse decline and relatively low prevalence of *T*. *brucei s*.*l*. in cattle.

## Introduction

In East and Southern Africa, tsetse flies (*Glossina* spp) transmit *Trypanosoma brucei rhodesiense*, which causes Rhodesian human African trypanosomiasis (r-HAT). Tsetse also transmit *T*. *congolense*, *T*. *vivax* and *T*. *brucei*, the causative agents of animal African trypanosomiasis (AAT) in livestock.

*Trypanosoma brucei s*.*l*., *T*. *congolense* and *T*. *vivax* can circulate in transmission cycles involving livestock or wild mammals [1]. The extensive conservation areas of East and Southern Africa that support tsetse, as well as wildlife, can therefore be foci for r-HAT and AAT. At the interface of wildlife and livestock areas, there is potential for trypanosomes to shift from a wildlife- to a livestock-dominated cycle of transmission [1]. Although existing r-HAT foci are often associated with wildlife areas, the importance of cattle as reservoirs at the wildlife-livestock interface is unclear [1].

There are few studies that address the role of cattle in r-HAT transmission in wildlife-livestock interface areas. In 2007, Kaare et. al. [2] suggested that r-HAT could be re-emerging in Serengeti District, Tanzania, based on surveys of cattle adjacent to the Serengeti National Park in 2001, where they found 5.6% of cattle positive for *T*. *brucei s*.*l*. DNA and approximately 1% of 518 cattle sampled as positive for *T*. *b*. *rhodesiense* DNA.

With approximately 1.4 million people living at moderate to high risk of *T*. *b*. *rhodesiense* in East and Southern Africa [3], there is a need to identify appropriate control measures that can reduce the risk of trypanosomiasis for both people and cattle living near wildlife areas. Previous modelling has indicated that insecticide-treated cattle could offer an effective method of control, particularly for r-HAT [4], but modelling has not been extended to consider wildlife-livestock interface areas.

We previously found that numbers of tsetse caught in traps declined by >90% across a wildlife-livestock interface in Serengeti District, Tanzania, with no tsetse being caught >5 km into farming areas [5]. We argued that this was due, in part, to reduced availability of habitat suitable for tsetse. This is likely to be typical of other r-HAT foci in and near wildlife areas, where increasing human and livestock densities lead to a reduction in tsetse habitat. However, the effect of habitat did not fully explain the change in tsetse abundance [5]. We obtained contemporary evidence that livestock farmers were frequently treating their cattle with pyrethroids, insecticides known to be highly effective against tsetse [6]. This raised the possibility that a sufficient proportion of cattle was being treated with insecticide to result in a reduction of the density of tsetse populations, and hence trypanosome infections.

We aimed to examine whether the presence of insecticide-treated cattle could be a contributing factor to the observed decline in tsetse. We used a combination of data collection and mathematical modelling to assess the impact of such a decline in tsetse on the transmission of trypanosomes in cattle at the interface between wildlife and livestock populations.

## Methods

### Ethics statement

Cattle sampling involved collection of venous blood and hair samples (procedures classified as 'mild' under UK Home Office regulations). Discussions regarding the veterinary sampling were undertaken with key administrative and community leaders to inform communities of the overall study and mobilise households to participate. Animals were sampled by veterinarians or trained paraveterinary workers. Jugular blood samples (10ml) were collected in sterile vacutainers and hair samples collected using a safety razor. The animals were restrained appropriately to minimise the time and distress involved in the process of sample collection. All sampling was undertaken under the supervision of a veterinarian. Ethical approval for this work was obtained from the SRUC Animal Experiments Committee and the Commission for Science and Technology (Costech) in Tanzania (permit number 2016-33-NA-2014-233).

### Study site

Our study site comprised the Serengeti National Park, adjacent game reserves and farming areas (S1 Fig). Farming areas are used predominantly for livestock grazing and crop production, with approximately 30 cattle/km^2^ [7].

The study site supports three species of tsetse–*G*. *swynnertoni*, *G*. *pallidipes* and *G*. *brevipalpis* [5]. The Serengeti area is an historic r-HAT focus [8]. Since the last outbreak in 2000/2001, during which at least 20 cases were reported in local populations and tourists [9,10], sporadic cases continue to occur [3].

### Tsetse surveys

We carried out surveys during February, June-July and October 2015 along four transects from 5 km inside wildlife areas, to 10 km into farming areas (S1 Fig). During each survey, we set a total of 72 odour-baited Nzi traps, 38 inside wildlife areas and 34 outside, and emptied traps each day for three consecutive days, recording the sex and species of tsetse. Full details of the survey method are provided in Lord et al. (2018) [5].

We caught fewer than 100 *G*. *brevipalpis* during the study, so our analyses focussed on *G*. *pallidipes* and *G*. *swynnertoni*. Since daily numbers (*y*) of tsetse caught per day in traps were overdispersed, we transformed the data to log_10_(*y* + 1) before calculation of average counts per trap.

During 2016 we carried out additional trapping inside wildlife areas, up to 10 km from the boundary, to catch sufficient numbers of tsetse to provide a robust estimate of the prevalence of *T*. *congolense* savanna and *T*. *brucei s*. *l*. in tsetse. *T*. *congolense* presence was used as a proxy for AAT, being more prevalent than *T*. *vivax* in the study area [2].

During each survey in 2015 and 2016, we transported tsetse flies, preserved in ethanol in individual tubes, to the Liverpool School of Tropical Medicine and processed them for the detection of trypanosome DNA (S1 Text).

### Livestock surveys

We carried out a cross-sectional livestock survey, in villages <5 km from the wildlife boundary, during July-August 2016. A total of 750 cattle were selected, from 48 herds, using a stratified selection method (S1 text). For each sampled animal, we collected blood from the jugular vein into Paxgene DNA tubes (Qiagen), and recorded details of the animal’s age, sex and any treatments given in the last six months. We asked farmers the date the animals were last treated with insecticide and the method of application. Blood samples were tested by PCR for the presence of *T*. *brucei* and *T*. *congolense* DNA (S1 Text).

In addition to asking farmers about use of insecticides, we also analysed hair for the presence of pyrethroids. Using disposable razors, we collected hair samples (0.04 g/animal) from the flank of four randomly-selected cattle within each herd, giving a total of 176 samples, which were sealed individually in foil bags. Cypermethrin and alpha-cypermethrin from each sample were extracted in acetone and assayed by gas chromatography-mass spectrometry (GC-MS) (S1 Text). This method can detect the presence of insecticide at 7 days post application, but not at 14 days [11].

### Data summary

We calculated the prevalence, and exact binomial 95% confidence intervals, for *T*. *brucei s*. *l*., *T*. *brucei rhodesiense* and *T*. *congolense* in cattle and tsetse as the percentage of individuals testing PCR positive for each trypanosome species and subspecies. For tsetse, this prevalence includes infected flies that might not be infectious.

To estimate the possible daily probability of mortality for adult tsetse attributable to insecticide-treated cattle, we assumed that any given tsetse fly contacts a vertebrate host either every two or every three days [12]. We then estimated the proportion of cattle treated, using information from hair sample analysis and farmer responses to questions. We divided this proportion by the duration of the feeding cycle, assuming that a fly would die from contacting any host testing positive for insecticide [6]. Under the hypothesis that cattle were treated with insecticide, we could not estimate the proportion of bloodmeals from cattle–because, by assumption, those that had fed on treated cattle would not be caught for analysis. We therefore made the assumption that cattle were the only source of bloodmeals in farming areas [13].

### Modelling tsetse population dynamics across the wildlife-livestock interface

To estimate the additional tsetse mortality in farming areas, we developed a spatially-explicit model of tsetse population dynamics and fitted the model to the tsetse catch data.

We describe changes in numbers of pupae (*P*) and adult tsetse (*A*) in space and time using two recursion equations on a lattice (S2 Text). Parameters used are described in Table 1.

**Table 1:** Parameters and values used in the model of tsetse population dynamics. Values are per 0.25 days. Each cell in the area modelled is a square of side 500 m.

Notation	Description	Value	Range	Reference
*l*	Probability female tsetse larviposits	0.025	0.02–0.031	[14,15]
*β*	Probability pupa emerges as an adult	0.008	0.005–0.0075	[15,16]
*δ*	Pupal density-dependent mortality coefficient	Fitted	10−5.60–10^−4.65^	NA
*μ*_*P*_	Pupal probability of mortality	0.0015	0.000625–0.0025	[15,17]
*μ*_*B*_	Adult baseline probability of mortality	0.0075	0.0025–0.0075	[18]
*a*	Adult diffusion coefficient	0.25	0.1–0.5	[19]
*μ*_*F*_	Adult additional probability of mortality in farming areas	Fitted	0.0075–0.125	NA

Reflecting boundaries were used in the lattice so that for cells at the edge of the lattice, numbers of tsetse moving in were equivalent to those leaving. Each day, in each cell *i*,*j* a proportion *a* of adult tsetse diffuse into adjacent cells. Adult females, assumed to be half the population, produce pupae with probability *l*. Adults die with probability *μ*_*B*_. Pupae emerge as adults with probability *β* and are subject to density-independent (*μ*_*P*_) and density-dependent (*Pδ*) deaths. In addition to the baseline mortality, adults present in cells designated as ‘farming’ areas are subject to an additional mortality (*μ*_*F*_) to represent insecticide use and habitat degradation.

We carried out a sensitivity analysis (S3 Text), to quantify how the modelled decline in tsetse density across the wildlife-livestock interface was influenced by model parameter values. We then fitted the model to observed tsetse abundance data using nonlinear least squares regression implemented with the Levenberg-Marquardt Algorithm, accounting for uncertainty in parameter values (S3 Text).

### Modelling trypanosome transmission dynamics across the wildlife-livestock interface

To quantify the effect of tsetse population decline on trypanosome prevalence in cattle in the interface area, we extended the tsetse model to include trypanosome transmission (S2 Text).

In addition to tsetse population dynamics described above, adult tsetse in each cell progress through susceptible teneral (juvenile unfed) (*S*_*V*_) to either susceptible non-teneral (*G*_*V*_), or exposed (*E*_*1V*_ *–E*_*3V*_) and then infectious (*I*_*V*_) classes. Instead of having a fixed-time for the incubation period for a trypanosome in a tsetse, or assuming that the incubation period is exponentially distributed, we model three exposed classes as per [20], assuming an Erlang distributed waiting time for the extrinsic incubation period [21]. Hosts in each cell progress through susceptible (*S*_*H*_), exposed (*E*_*H*_), infected/ infectious (*I*_*H*_) and recovered (*R*_*H*_) classes. We assumed that host populations do not move, and host birth and death rates are equal.

Due to uncertainty in parameter values (Table 2) for trypanosome transmission, we quantified the potential effect of the tsetse population decline on transmission across the interface, by running a sensitivity analysis, first without increased tsetse mortality (S3 Text). From the sensitivity analysis, we then selected combinations of parameter values that produced trypanosome prevalence in tsetse at equilibrium within the range observed in our study site for *T*. *brucei* and *T*. *congolense*. We ran the model using the selected parameter combinations and including an additional tsetse mortality, the value of which we obtained from fitting the model of tsetse population dynamics to observed tsetse abundance.

**Table 2:** Parameters and values used in the trypanosome transmission model. See Table 1 for tsetse population dynamics parameters.

Notation	Description	Value	Range*	Reference
*β*_*H*_	Host daily probability of birth	0.0003	NA	NA
*μ*_*H*_	Host daily probability of mortality	0.0003	NA	NA
*α*	Daily probability of tsetse feeding		1/3–1/2	[22]
*p*_*S*_	Probability of teneral tsetse acquiring trypanosome infection given bite on an infected host		0–0.5	[23–26]
*p*_*G*_	Probability of non-teneral tsetse acquiring trypanosome infection given bite on an infected host		0–0.1	[23,25,26]
*σ*_*V*_	Proportion of infected tsetse that become infectious per day		1/30–1/15	[21]
*p*_*H*_	Probability of host acquiring trypanosome infection given bite from infectious tsetse		0.2–0.8	NA
*γ*	Probability of recovered host becoming susceptible per day		1/100–1	NA
*σ*_*H*_	Proportion of exposed/ infected hosts that become infectious per day		1/15–1/5	[27,28]
*φ*	Proportion of infected hosts that recover per day		1/100–1/25	[27,28]

*Values used for both *T*. *brucei* and *T*. *congolense*

The tsetse population dynamics and trypanosome transmission models, plus code to produce the associated figures can be accessed at https://github.com/jenniesuz/tsetse_wli.git.

## Results

### Observed tsetse decline across the wildlife-livestock interface

**Fig 1:**
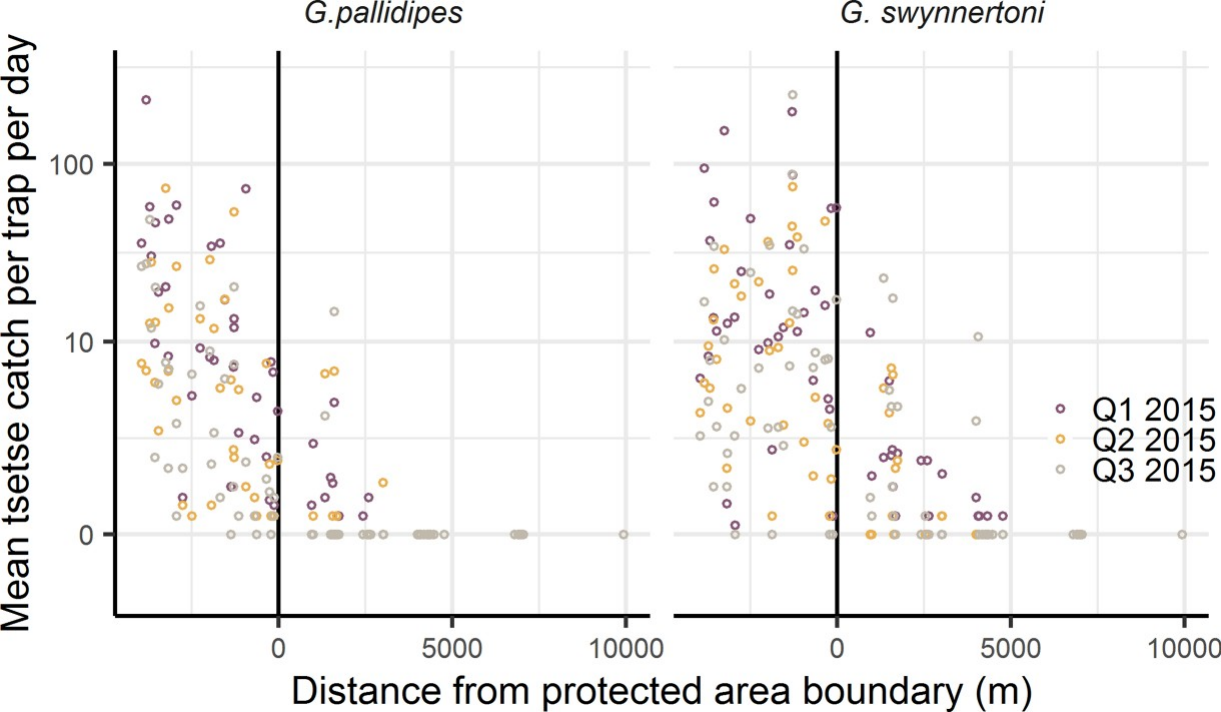
Mean numbers of tsetse caught across the wildlife-livestock interface by season and species during 2015.

Mean daily numbers of *G*. *pallidipes* and *G*. *swynnertoni* caught per trap declined to zero by 5 km outside wildlife areas in the second and third quarterly surveys of 2015, as also observed in the first survey in February 2015 (Fig 1, [5]). Across all three surveys in wildlife areas, >99% of traps caught at least one tsetse, whereas in farming areas 58% of traps did not catch any flies.

### Observed trypanosome prevalence in tsetse and cattle

During 2015 and 2016 we caught 5986 tsetse, which were tested for the presence of trypanosome DNA. Only 4% of flies sampled during 2015 were from farming areas. Both *T*. *congolense* and *T*. *brucei s*.*l*. were detected and two flies from wildlife areas tested positive for *T*. *b*. *rhodesiense* (Table 3). Of the 750 cattle sampled in 2016, none was positive for *T*. *b*. *rhodesiense* DNA and *T*. *brucei s*.*l*. prevalence was 2.3% compared to 16.7% for *T*. *congolense* (Table 3).

**Table 3:** Prevalence of trypanosome species in tsetse and cattle. Prevalence defined as the percentage of hosts or vectors testing positive for the presence of DNA for the respective species: 95% confidence intervals in parentheses.

		Prevalence (%)		
	Number sampled	*T*. *brucei rhodesiense*	*T*. *brucei s*.*l*.	*T*. *congolense*
**Tsetse**	5986	0.03 (0.004–0.121)	1.25 (0.09–1.57)	5.34 (4.79–5.94)
*G*. *pallidipes*	3246	0.031 (0.0008–0.18)	1.69 (1.28–2.20)	4.99 (4.27–5.80)
*G*. *swynnertoni*	2735	0.037 (0.0009–0.20)	0.73 (0.45–1.13)	5.92 (5.07–6.87)
*G*. *brevipalpis*	5	0 (0–52.2)	0 (0–52.2)	20.00 (0.51–71.64)
**Cattle**	750	0 (0–0.49)	2.3 (1.3–3.6)	16.7 (14.1–19.5)

### Insecticide use

Of the 48 livestock owners questioned about insecticide use, 67% reported treating at least some of their cattle with a pyrethroid within the previous month and 16% reported treating within the previous week. Chemical analyses of hair samples collected at the time of the survey showed that 18% of 176 individual cattle and 27% of 48 herds had detectable levels of alpha cypermethrin or cypermethrin (ranging from 1.6 μg/g to 1278 μg/g), indicating treatment within approximately 7 days previously.

If we assume a three-day feeding cycle, and that 16% of cattle are treated weekly, tsetse probability of mortality from insecticide-treated cattle would be approximately 0.05 per day. If we assume a two-day feeding cycle and that 27% cattle are treated, the probability of mortality from insecticide would be approximately 0.14 per day.

### Simulating tsetse population dynamics across the wildlife-livestock interface

Catches of both *G*. *pallidipes* and *G*. *swynnertoni*, across all seasons, declined to zero by 5 km outside wildlife areas (Fig 1). We therefore fitted the tsetse population dynamics model to mean tsetse catches per trap per day including both species and across all seasons.

**Fig 2:**
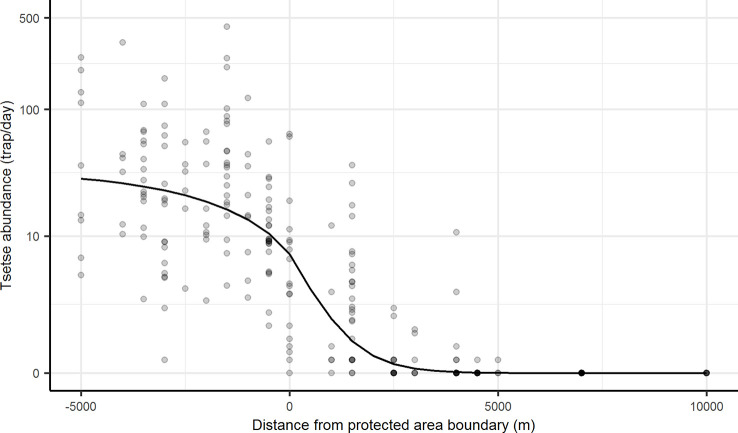
Modelled decline in tsetse abundance across the wildlife-livestock interface. Model fitted by nonlinear least squares regression to mean daily tsetse caught per trap across three surveys in 2015. Negative distances on *x* axis indicate inside wildlife areas where no additional mortality was modelled. The *y* axis is on log_10_ scale. Darker points indicate samples from multiple traps at the same distance.

Using the parameter values in Table 1, the best fit additional daily probability of adult mortality (*μ*_*F*_) was 0.15 per day (S1 Table, Fig 2). Of the fixed parameters, daily dispersal distance (*a*) and daily probability of larviposition (*l*) had the biggest influence on the relative density of tsetse 1 km inside farming areas, compared to density 5 km inside wildlife areas, with PRCC > 0.5 and < -0.5, respectively (S2 Fig and S3 Fig). Depending on values for the daily probability of larviposition and dispersal, fitted values for additional daily probability of mortality varied between 0.08 and 0.29 (S1 Table).

### Simulating trypanosome transmission across the wildlife-livestock interface

**Fig 3:**
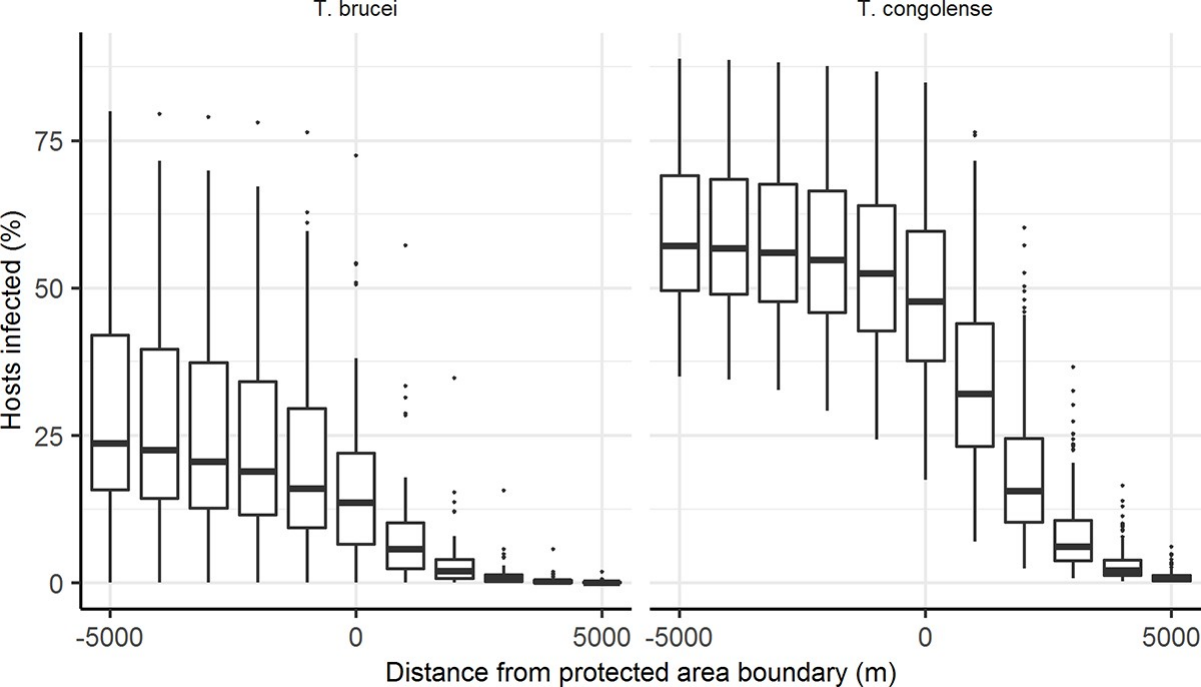
Modelled decline in trypanosome prevalence across the wildlife-livestock interface. Assuming an additional probability of tsetse mortality/day in farming areas of 0.15 as per model fits to the observed tsetse data. The solid horizontal line in each boxplot shows the mean output from model runs using combinations of parameter values from sensitivity analysis that could explain the observed tsetse prevalence and hinges represent 25^th^ and 75^th^ percentiles.

Of the parameters detailed in Table 2, host incubation, host probability of infection and probability of recovery had the biggest effect on prevalence of trypanosomes in hosts, while the proportion of infected hosts that recover per day, and host-to-vector transmission probabilities had the biggest effect on prevalence of trypanosomes in vectors (S4 Fig and S5 Fig). From sensitivity analysis, of 1000 simulations with different parameter values, 138 had tsetse prevalence within the confidence intervals of that observed for *T*. *brucei s*.*l*. and 150 for *T*. *congolense*. Using these remaining parameter combinations, with the estimated additional mortality, *T*. *brucei* prevalence in hosts, averaged across all simulations, was 9.8% at 1 km from wildlife areas, declining to 4.0% by 2 km outside of wildlife areas, while *T*. *congolense* prevalence was 45.1% at 1 km outside of wildlife areas and 27.7% by 2 km (Fig 3).

## Discussion

We report that farmers in Serengeti District use pyrethroid-based insecticides at rates sufficient to impact tsetse populations. Our results support the findings of Ngumbi et al. [29] who reported the use of pyrethroids by farmers in Pangani, Myomero and Korogwe districts of Tanzania. To our knowledge, however, our study is the first to report farmer-led tsetse control, co-incident with tsetse decline and relatively low prevalence of *T*. *brucei s*.*l*. in cattle. There are other examples of insecticide-treated cattle being used to control tsetse and trypanosomiasis, but these were implemented by commercial ranches or received strong support from government institutions or donors [30–33]. Farmers in this region were using only cypermethrin or alpha cypermethrin; no other insecticides were reported by livestock farmers [11]. The scale of use beyond the study area and in other livestock production systems, and the drivers for individual farmers to choose to treat their cattle deserve further scrutiny and are the subject of further investigations.

Coupling questionnaires concerning insecticide use with validation via hair sample analysis would be beneficial in further investigations. Questionnaires are useful for gathering information on use, but issues with product labelling, including language translation, can result in inadequate application of insecticide [34], which may not be identified through a questionnaire approach. Quantification of insecticide on hair samples provided reassurance that use reported by farmers accurately reflected insecticide administration within the past week. The use of gas chromatography-mass spectrometry for analysis of livestock hair samples is expensive and a more cost-effective method for quantifying insecticide concentrations would aid larger-scale assessments of actual use in future studies.

Modelling suggests that in areas of relatively high cattle density, such as our study site, where the majority of tsetse blood meals are from cattle, modest use of insecticide-treated cattle by livestock farmers can reduce the role of cattle in *T*. *b*. *rhodesiense* transmission despite the presence of high tsetse densities in adjacent wildlife areas. However, our data suggest that treating cattle with pyrethroids may be less effective against AAT [4] and *T*. *congolense* in particular. Farmers at the boundary of wildlife areas may also treat their animals with trypanocides (although notably trypanocide use may be compromised due to resistance [35]). *T*. *brucei s*.*l*. and *T*. *b*. *rhodesiense* prevalence in cattle in Serengeti District was documented during 2001 coincident with cases of r-HAT [2]. The *T*. *brucei s*.*l*. prevalence in our study was 1.25% (0.09–1.57) compared with 5.6% (3.78–7.94) estimated by Kaare et al. [2] and this trend would therefore suggest that there may have been a decrease in risk in this area over time.

Our modelling involved several assumptions. We assumed that there was no overall change in tsetse population and trypanosome prevalence in wildlife areas over time. We did not account for seasonal changes in wild host movement which may influence trypanosome prevalence in adjacent wildlife areas and therefore risk of infection in cattle. Nor did we account for trypanocide use, heterogeneity in insecticide-treated cattle use, or habitat quality in farming areas. These are also likely important factors driving trypanosome prevalence. It was not possible to account for all potential sources of heterogeneity in the transmission system, nor was it necessary for the aim of our modelling. Using the trypanosome transmission model, we aimed to quantify the potential effect of the estimated increase in tsetse mortality on trypanosome transmission across the wildlife-livestock interface, in the absence of other influencing factors. The modelling results indicate that even without trypanocide use by farmers, trypanosome prevalence in cattle would be reduced because of increased tsetse mortality.

We could not disentangle the relative contributions of habitat degradation and insecticide-treatment of cattle to increased tsetse mortality. This would require further studies using controlled trials across areas differing in habitat and land use. Our study does, however, extend the modelling carried out by Hargrove et al. [4] in being spatially-explicit and considering an interface context. A better understanding of the relative contribution of habitat degradation to tsetse decline at wildlife-livestock interface areas would help to identify where and when insecticide-treated cattle would be most effective. Treatment of cattle with insecticide offers a cost-effective method of tsetse control [36] and in East Africa the risk of both tick- and tsetse-borne diseases of livestock provides a strong incentive for livestock farmers to treat their cattle regularly [37]. Effective control of savanna tsetse requires interventions conducted over large (>100 km^2^) areas [38]. This is possible for large commercial ranches [30,31] but much more difficult to implement and sustain with small-scale livestock farmers without co-ordination and financial support from donor or government agencies. Our findings, however, provide evidence that small-scale farmers can be enabled to control r-HAT.

Cost-benefit analyses have not included newer methods that have been developed to reduce tsetse and trypanosome transmission, which involve using odour repellent collars on livestock in combination with insecticide-treated targets baited with attractant odours [39]. Comparative analyses of such methods and insecticide-treated livestock would be of benefit to inform their relative utility in tsetse control efforts [36].

If insecticide-treated cattle is to be a realistic and sustainable means of tsetse and trypanosomiasis control, the potential environmental effects of the insecticides must be considered. The insecticide reported to be widely used in this study area (alpha cypermethrin) has a half-life of approximately 1 week on vegetation or in soil [40], and in terms of contamination of dung, Vale *et al*. demonstrated that when insecticides are applied only to the areas where tsetse most frequently bite, death of dung beetles was reduced to negligible levels [41]. Potentially adverse environmental impacts could therefore be mitigated through informed restricted application of pyrethroids to cattle [6].

For insecticide-treated cattle to be deployed at scale and, importantly, sustainably, a critical aspect to understand is what the drivers are that motivate farmers in Serengeti to adopt this strategy. For example, if ticks and tick-borne diseases are a major stimulus, then options that mitigate against insecticide resistance in the tick vector would be a priority to ensure sustainability. Understanding the underlying social, economic and political drivers of this phenomenon could therefore potentially lead to the elusive goal of sustainable and cost-effective control of trypanosomiasis in east and southern Africa.

## Supporting information

S1 Fig Study site location. (DOCX)

S2 Fig Scatter plots showing the relationship between model parameters and output. (DOCX)

S3 Fig Partial rank correlation coefficient for each parameter in the tsetse population dynamics model. (DOCX)

S4 Fig Results of sensitivity analysis for the trypanosome transmission model. (DOCX)

S5 Fig Partial rank correlation coefficients for the trypanosome transmission model. (DOCX)

S1 Text Additional methods. (DOCX)

S2 Text Model equations. (PDF)

S3 Text Model sensitivity analysis and model fitting. (DOCX)

S1 Table Fitted model parameter values. (DOCX)
