# Supplementary material for: Assessing the effect of insecticide-treated cattle on tsetse abundance and trypanosome transmission at the wildlife-livestock interface in Serengeti, Tanzania
Source: PLoS Negl Trop Dis. 2020 Aug 25;14(8):e0008288. doi: 10.1371/journal.pntd.0008288 (PMC7473525; doi:10.1371/journal.pntd.0008288)

**Supplementary Figure 1. Study site location.** Inset: Tanzania protected areas – rectangle depicting study area along northern border of the Serengeti National Park. Main figure: protected areas in the study comprised Grumeti and Ikorongo Game Reserves, Ikoma Wildlife Management Area and the Serengeti National Park. Crosses represent locations of cattle herds that were sampled and open circles indicate tsetse trapping sites.


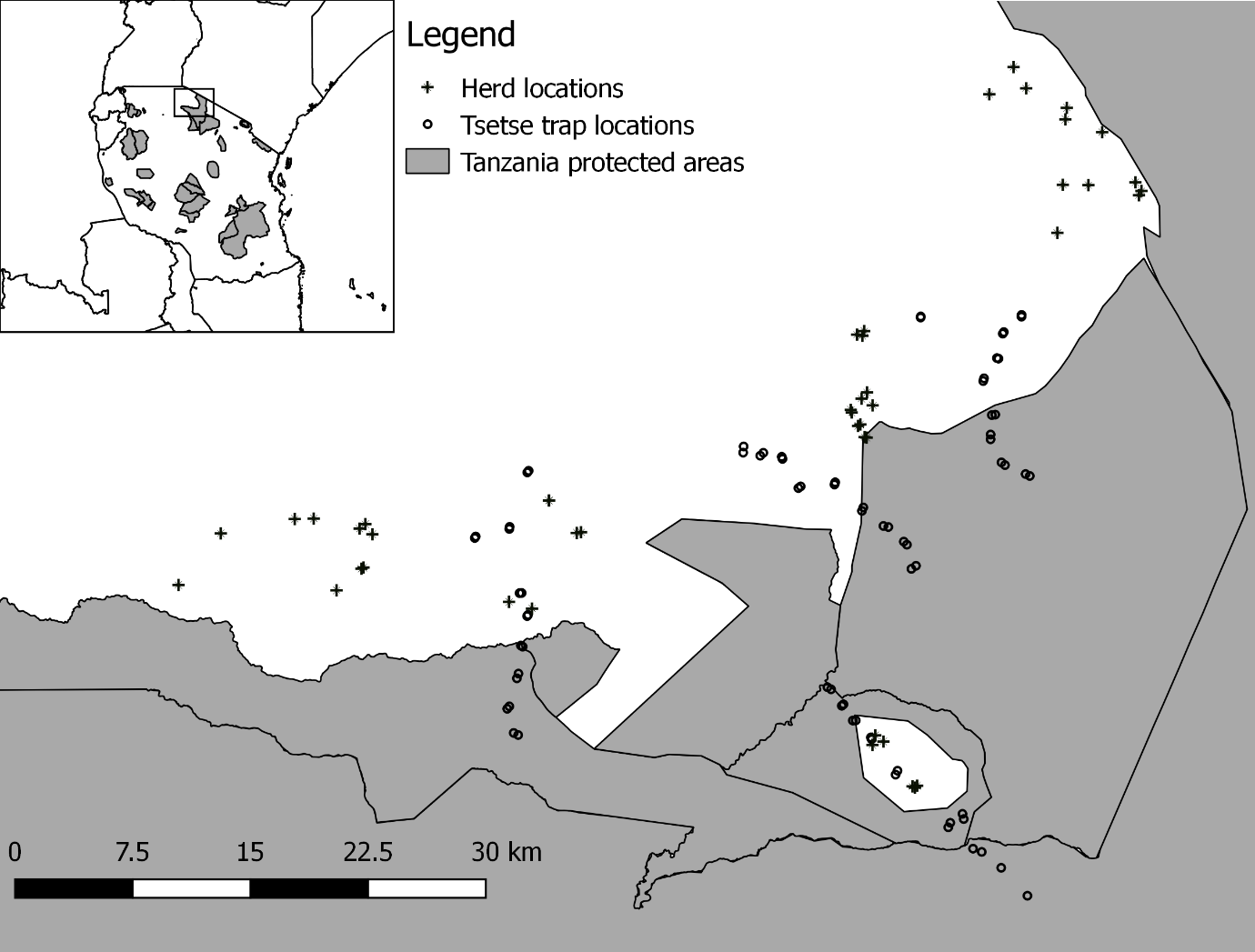

Supplement: S1 Fig — (DOCX) [file pntd.0008288.s001.docx]
