# Supplementary figures and images for: Assessing the effect of insecticide-treated cattle on tsetse abundance and trypanosome transmission at the wildlife-livestock interface in Serengeti, Tanzania
Source: PLoS Negl Trop Dis. 2020 Aug 25;14(8):e0008288. doi: 10.1371/journal.pntd.0008288 (PMC7473525; doi:10.1371/journal.pntd.0008288)

**Scatter plots showing the relationship between model parameters and output.**


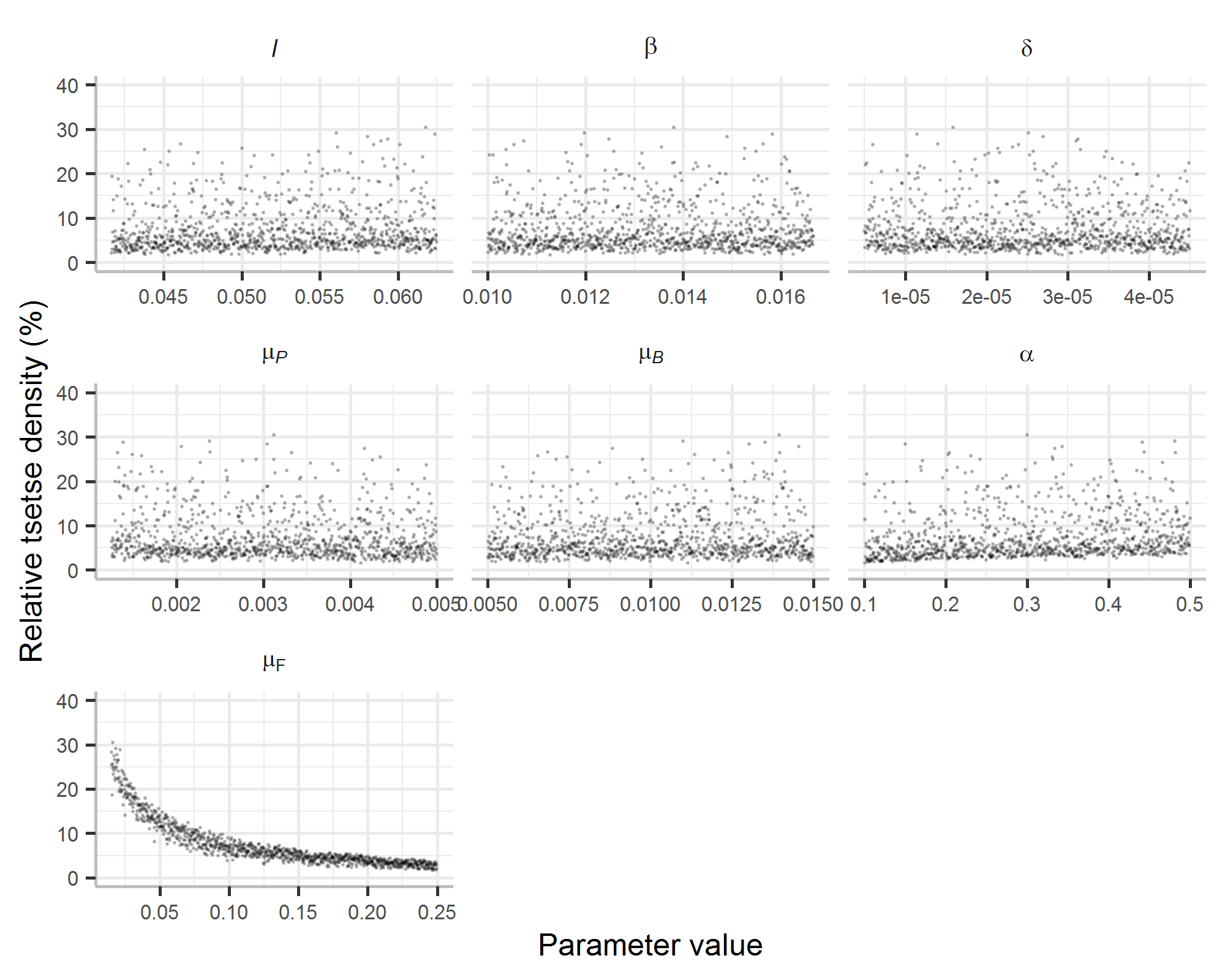

Supplement: S2 Fig — (DOCX) [file pntd.0008288.s002.docx]
