# Supplementary material for: Assessing the effect of insecticide-treated cattle on tsetse abundance and trypanosome transmission at the wildlife-livestock interface in Serengeti, Tanzania
Source: PLoS Negl Trop Dis. 2020 Aug 25;14(8):e0008288. doi: 10.1371/journal.pntd.0008288 (PMC7473525; doi:10.1371/journal.pntd.0008288)

**Partial rank correlation coefficient for each parameter in the tsetse population dynamics model.**


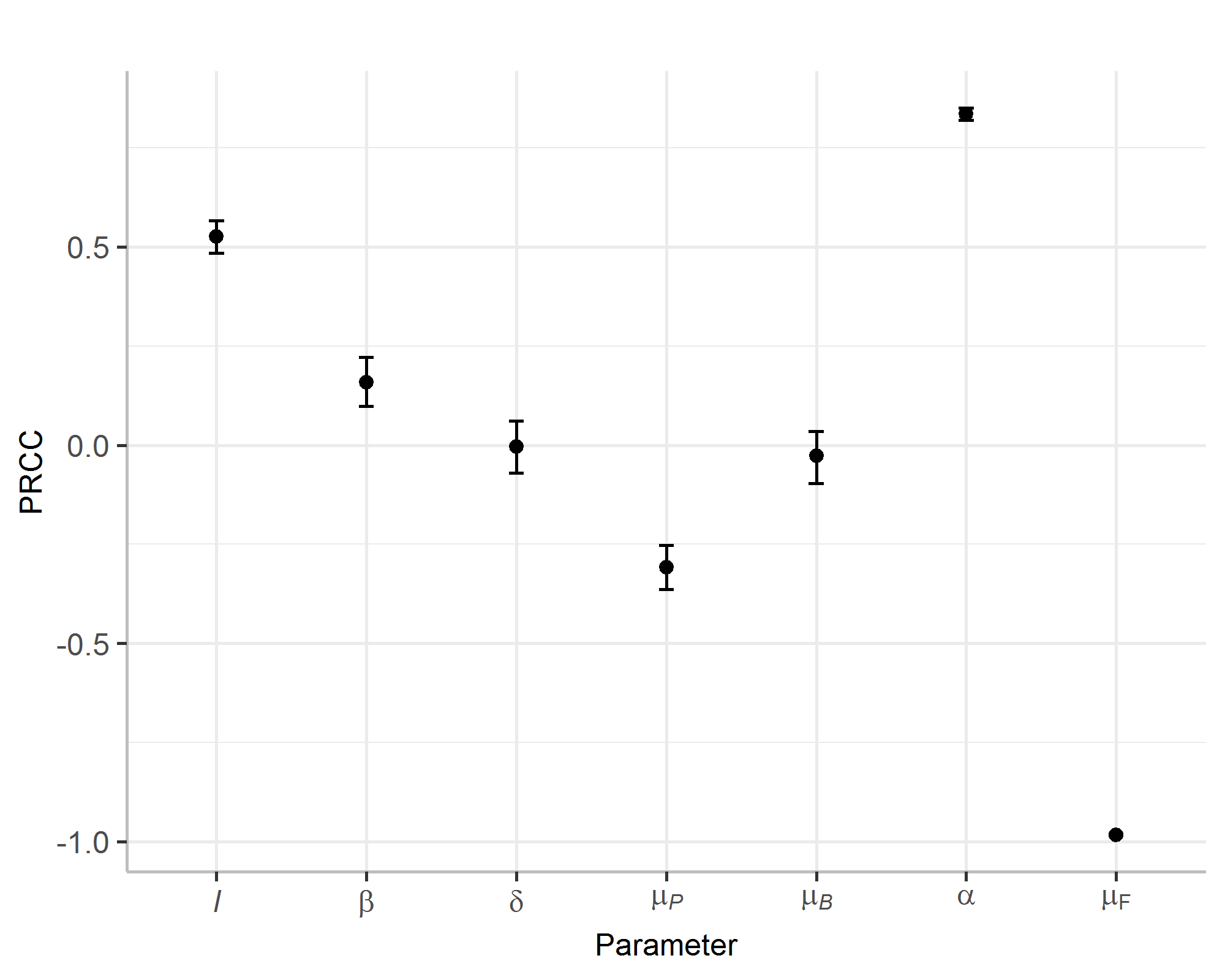

Supplement: S3 Fig — (DOCX) [file pntd.0008288.s003.docx]
