# Supplementary material for: Assessing the effect of insecticide-treated cattle on tsetse abundance and trypanosome transmission at the wildlife-livestock interface in Serengeti, Tanzania
Source: PLoS Negl Trop Dis. 2020 Aug 25;14(8):e0008288. doi: 10.1371/journal.pntd.0008288 (PMC7473525; doi:10.1371/journal.pntd.0008288)

**Results of sensitivity analysis for the trypanosome transmission model.** Host prevalence at equilibrium.

**
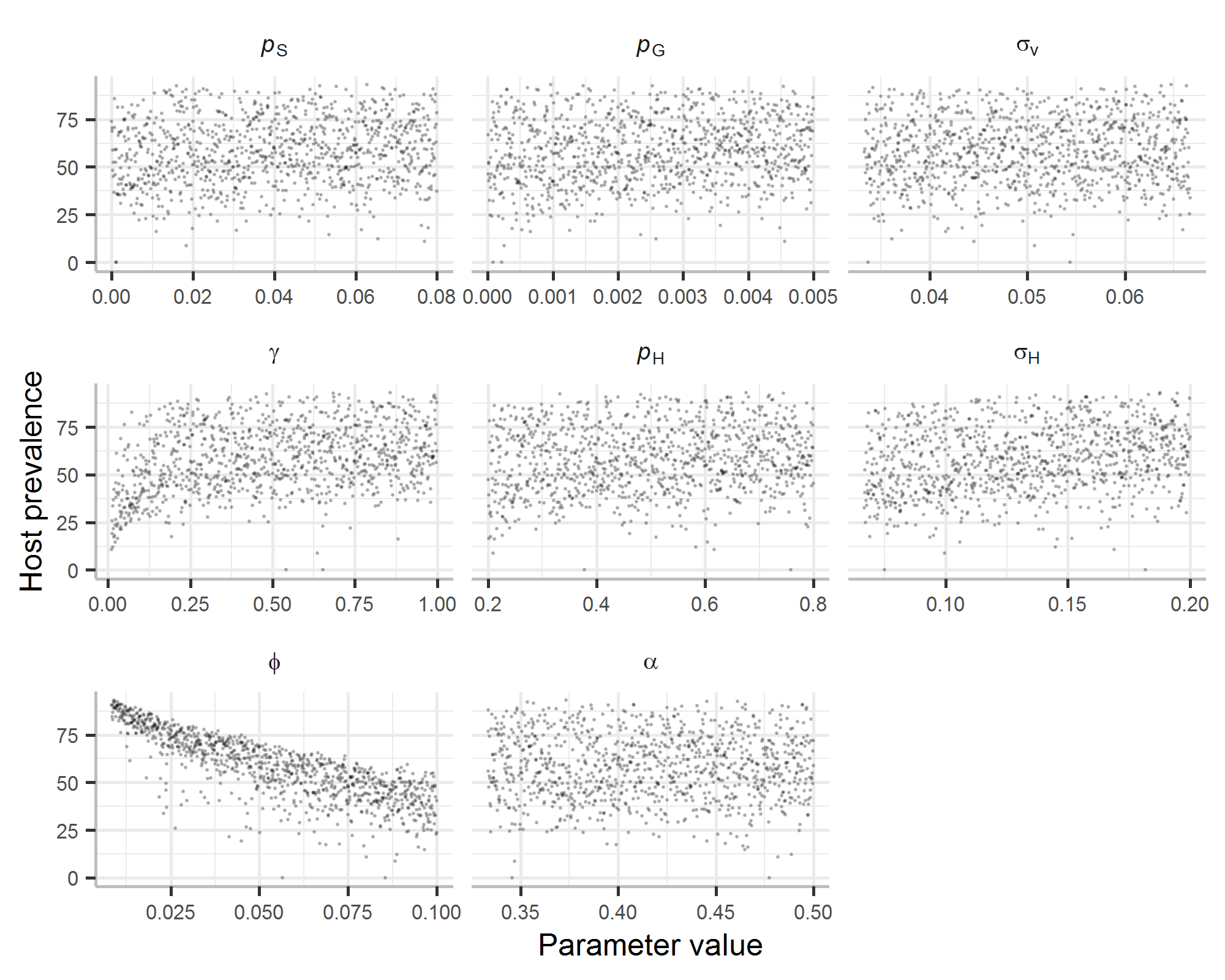
**

**Results of sensitivity analysis for the trypanosome transmission model.** Vector prevalence at equilibrium.


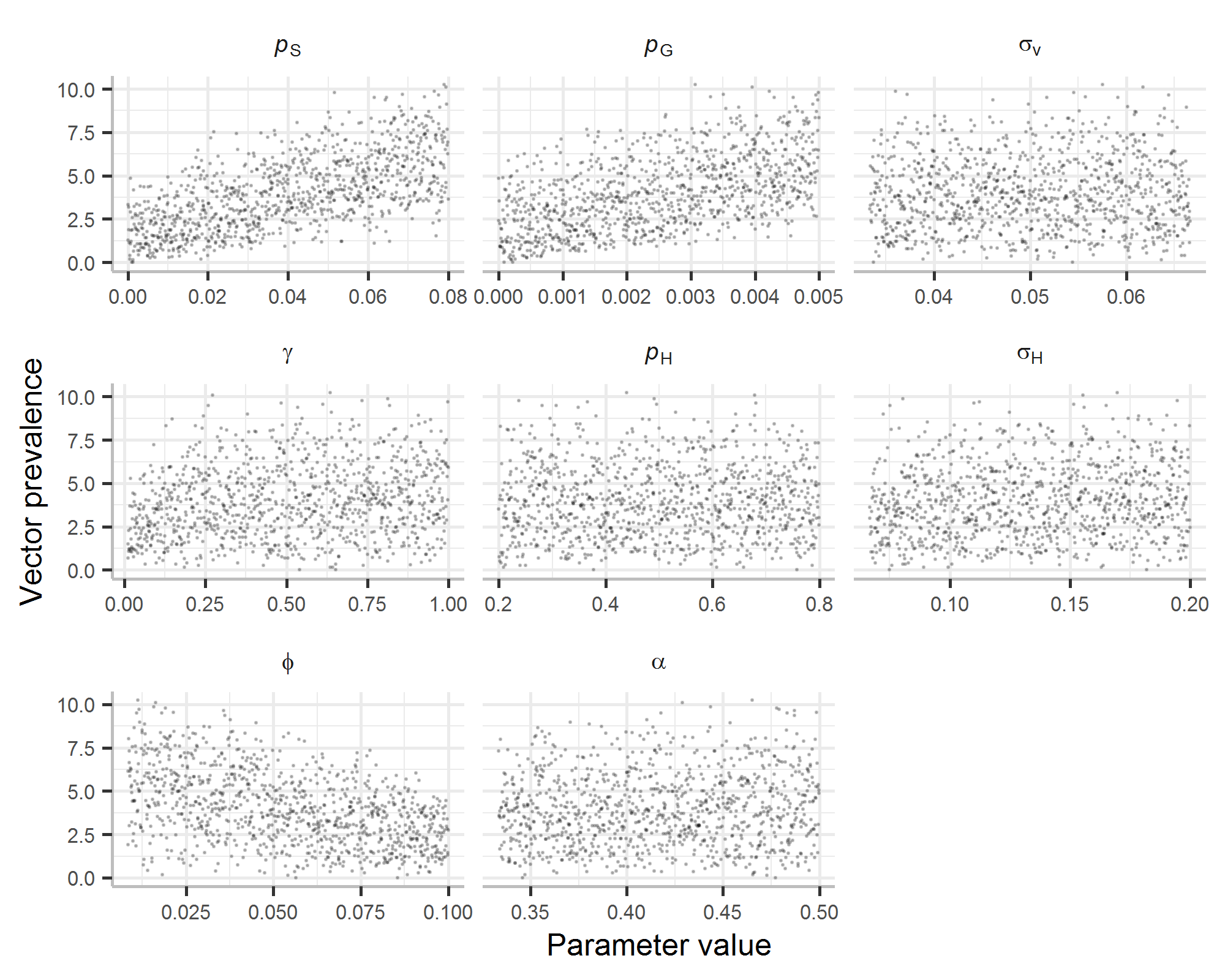

Supplement: S4 Fig — (DOCX) [file pntd.0008288.s004.docx]
