# Supplementary material for: Assessing the effect of insecticide-treated cattle on tsetse abundance and trypanosome transmission at the wildlife-livestock interface in Serengeti, Tanzania
Source: PLoS Negl Trop Dis. 2020 Aug 25;14(8):e0008288. doi: 10.1371/journal.pntd.0008288 (PMC7473525; doi:10.1371/journal.pntd.0008288)
Supplement: S5 Fig — (DOCX) [file pntd.0008288.s005.docx]

**Partial rank correlation coefficients for host prevalence as model output.**

**
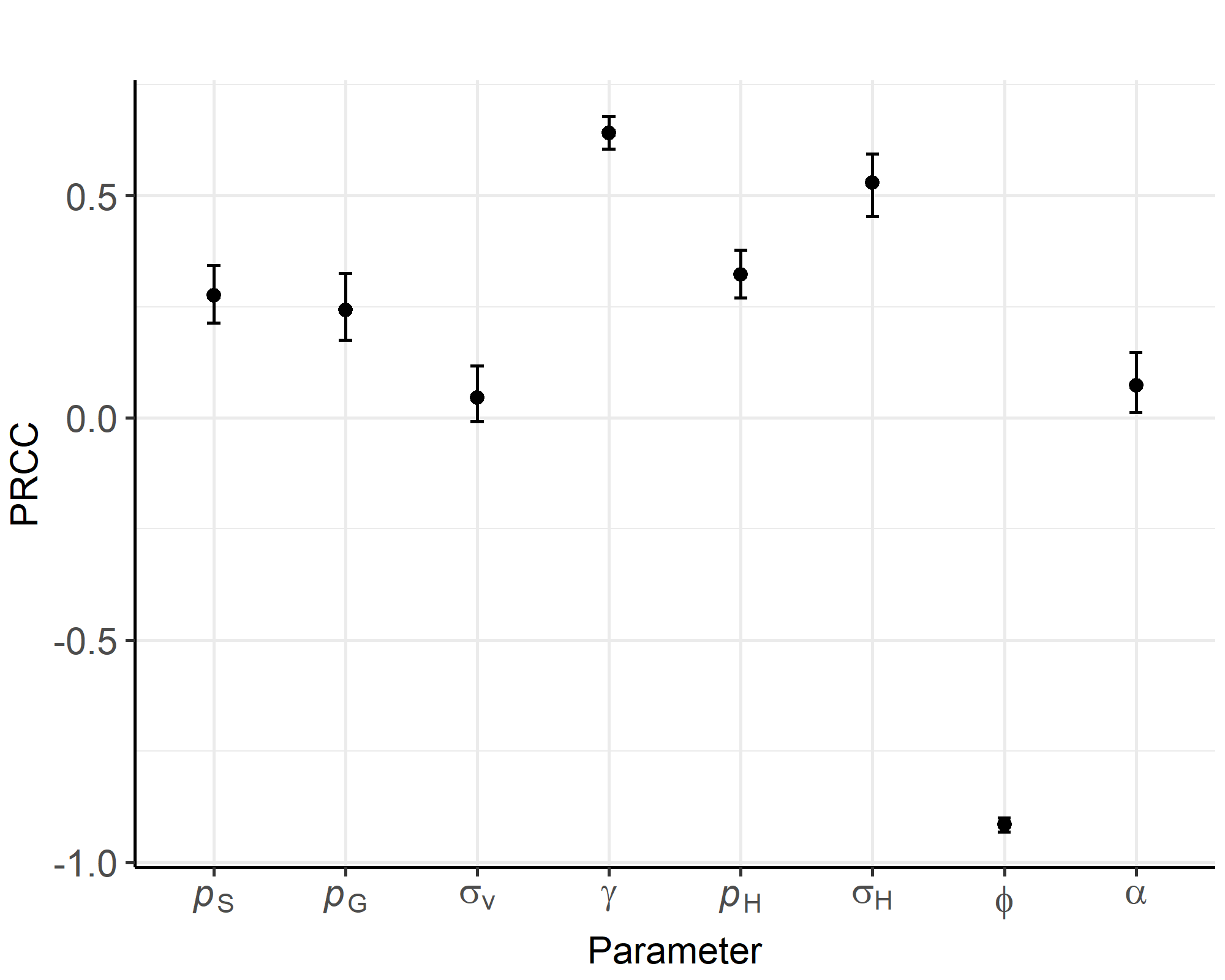
**

**Partial rank correlation coefficients for vector prevalence as model output.**

**
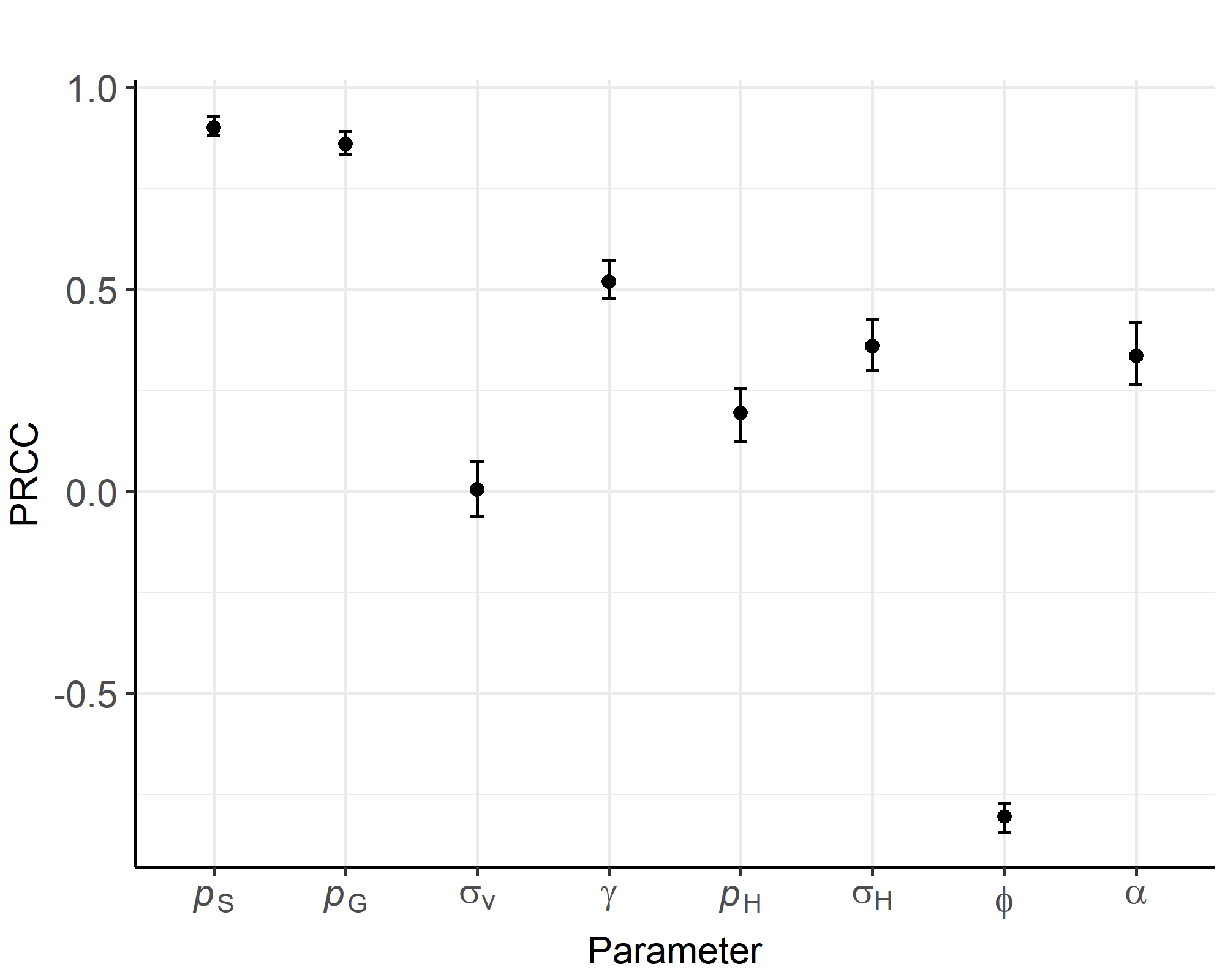
**
