# Supplementary material for: Assessing the effect of insecticide-treated cattle on tsetse abundance and trypanosome transmission at the wildlife-livestock interface in Serengeti, Tanzania
Source: PLoS Negl Trop Dis. 2020 Aug 25;14(8):e0008288. doi: 10.1371/journal.pntd.0008288 (PMC7473525; doi:10.1371/journal.pntd.0008288)
Supplement: S1 Text — (DOCX) [file pntd.0008288.s006.docx]

**Detection of *Trypanosoma brucei* and *T. congolense* DNA in tsetse flies**

To estimate the numbers of flies that would need to be collected to estimate trypanosome prevalence, we assumed a prevalence of 3% for *T. brucei,* including both immature and mature infections. Assuming 95% confidence and a margin of error of 0.5%, we required ~4500 flies. Similarly, to estimate a prevalence of 7% *T. congolense* ~2500 flies were required [1,2].

Whole tsetse flies preserved in 100% ethanol and DNA extracted using a Genejet Genomic DNA Purification kit (Thermo Fisher), following the protocol for mammalian tissue. Samples were homogenised and incubated at 56˚C overnight in a solution of Proteinase K and Digestive solution. RNase A was added, followed by lysis solution and 50% ethanol. Two wash steps using ethanol-based solutions were completed before the remaining DNA was eluted with a final volume of 200µl. Extracted DNA was stored at -4 degrees until it was ready for use in downstream applications.

Trypanosome DNA was detected by PCR using oligonucleotide primers [3] that target sequences in DNA satellite repeats specific to either *T. brucei* or *T. congolense.* From samples positive for *T. brucei*, primers were then applied that specifically amplify the SRA gene of *T. b. rhodesiense* [4] to test for the potential presence of the human-infective subspecies.

PCR reactions consisted of 9.5µl nuclease free water (Fisher), 1µl of forward and reverse primer (25mmol) and 12.5µl DreamTaq Green PCR master mix (2x) (Thermo Fisher). Amplifications were carried out in 25µl volumes with initial denaturation at 95˚C for 3 minutes, followed by 35 cycles of 95˚C for 30s, 55˚C for 45s and 72˚C for 30s with a final extension at 72˚C for 5 minutes. Amplified DNA products were confirmed with 1% agarose gel in 0.5× electrophoresis buffer and visualized under ultraviolet (UV) light after staining with 10ul/100ul PeqGreen (VWR). A 100 bp DNA ladder (Bioline) was used as the standard marker for comparison. Amplified products were cleaned up by adding 0.5 µl of Exo I (#M0293, NEB) and 1 µl of rSAP (#M0371, NEB) to 5 µl of PCR product. The samples were incubated at 37°C for 15 minutes, followed by 80°C for 15 minutes. Samples were then sent to a third party (Source Bioscience) for sequencing. The returned sequences were checked against known sequence in GenBank using BLAST.

**Livestock survey and sample processing**

Herds were selected using a stratified selection method. Of 18 villages in the target population, we randomly selected eight for study and in each we focussed on two sub-villages chosen at random. In each sub-village, we carried out cattle sampling and a questionnaire survey in three cattle-owning households. We obtained written consent from participating households. Up to 20 cattle were sampled per herd, or all animals for herds of less than 20. Only animals over six months old were included. Animals were marked with dye to avoid re-sampling, and, as far as possible, randomly selected. In total, we sampled 750 cattle from 48 herds, sufficient to detect a prevalence of 6% (*T. brucei* [2]) at 95% confidence and margin of error of 2.5%, and accounting for a clustered population with a design effect of 2.1 assuming mean animals sampled of 15 per herd (intracluster correlation coefficient estimated at 0.08 based on previous data).

Cattle blood was sampled via the jugular vein into Paxgene Blood tubes (Qiagen), which were stored at -20°C until processing. The Paxgene Blood DNA Kit (Qiagen) was used to isolate genomic DNA as follows: frozen Paxgene blood tubes were thawed at ambient temperature for approximately two hours. Each blood sample (8.5 ml) was mixed by inverting ten times before being transferred to a tube pre-filled with 25 ml of cell lysis buffer BG1. The solution was mixed by inverting five times before centrifuging at 2500 x *g* for five minutes. Supernatant was carefully discarded to leave a pellet before the addition of 5 ml Buffer BG2. The pellet was washed by vortexing for five seconds and then centrifuged at 2500 x *g* for three minutes. Supernatant was removed and 5ml of digestion buffer BG3, including reconstituted PreAnalytiX Protease, was added before vortexing at high speed for 20 seconds to dissolve the pellet. The tube was incubated in a water bath at 65°C for 10 minutes, and then vortexed for five seconds before the addition of 5 ml isopropanol (100%) and mixed by inverting 20 times to precipitate the DNA. Tubes were centrifuged at 2500 x *g* for three minutes, the supernatant removed and the tubes inverted on absorbent paper for one minute in order to remove maximal isopropanol. A volume of 5 ml of 70% ethanol was added to the tube and vortexed for one second before centrifuging at 2500 x *g* for three minutes. Supernatant was removed and 1 ml volume of resuspension buffer, BG4, was added to the dried pellet and the tube incubated in a water bath at 65°C for one hour, followed by overnight incubation at ambient temperature to dissolve DNA.

PCRs to detect *T. brucei* and *T. congolense* were carried out as described above for tsetse fly samples.

**Quantification of insecticide on cattle hair samples**

Each hair sample was weighed (0.05 - 0.15 g) and transferred to a glass tube, then washed with 4ml of acetone and sonicated for 15 minutes. The insecticide-acetone solution was then removed and placed into a fresh tube, then the wash and sonication step repeated with another 4ml of acetone and added to the previously removed 4ml. The 8ml of solution was evaporated then resuspended in 4 ml of acetone, split into two vials and evaporated again. One set of samples was then sent to the Natural Resources Institute for gas chromatography - mass spectrometry analysis and the other set kept for future analysis.

Samples were dissolved in 1 ml acetone (Pesticide Residue Grade, Fisher) and analysed by gas chromatography-mass spectrometry (GC-MS) on an Agilent 6890 GC coupled directly to an Agilent 5973 Mass Selective Detector (Agilent Technologies, Manchester, UK). The GC was fitted with a fused silica capillary column coated with DB5 (30 m x 0.125 mm i.d. x 0.125 μ film thickness; Agilent). Carrier gas was helium (1 ml/min) injection was splitless (300 °C) and the oven temperature was programmed from 60 °C for 2 min then at 10 °C/min to 300 °C and held for 10 min. The transfer line temperature was 250 °C, quadrupole 150 °C and ion source 230 °C.

Injections of 2 or 3 μl were made with an autosampler, and total ion current (TIC) monitored from m/z 30-450. Cypermethrin and alpha-cypermethrin were detected by single ion scanning at m/z 163 and 181 and verification of the mass spectrum where possible. Calibration curves of cypermethrin were run from 0.3-30 ng/μl and quantification was done by these external standards using *m/z* 163.

Cypermethrin gave four approximately equal-sized peaks ( 8 pairs of enantiomers) with peaks 1, 2 and 3 resolved but peaks 3 and 4 only partially resolved. Alpha-cypermethrin showed generally only peaks 1 and 3 with peak 3 predominating, corresponding to the two *cis* isomers, although in some samples peak 2 was also present. In some samples, tetracosane (24:H; 50 ng) was added as internal standard and quantification used the TIC for 24:H and m/z 163 for the cypermethrin, but this was found to be less reliable.

1. Mwambu P, Mayende J. Sleeping sickness survey in Musoma District, Tanzania III. Survey of cattle for evidence of T. rhodesiense infections. Acta Trop. **1971**; 28(3):206–210.

2. Kaare MT, Picozzi K, Mlengeya T, et al. Sleeping sickness - a re-emerging disease in the Serengeti? Travel Med Infect Dis. **2007**; 5(2):117–24.

3. Masiga D, Smyth A, Hayes P, Bromidge T, Gibson W. Sensitive detection of trypanosomes in tsetse flies by DNA amplification. Int J Parasitol. **1992**; 22(7):909–918.

4. Radwanska M, Chamekh M, Vanhamme L, et al. The serum resistance-associated gene as a diagnostic tool for the detection of Trypanosoma brucei rhodesiense. Am J Trop Med Hyg. **2002**; 67(6):684–690.
