# Supplementary material for: Assessing the effect of insecticide-treated cattle on tsetse abundance and trypanosome transmission at the wildlife-livestock interface in Serengeti, Tanzania
Source: PLoS Negl Trop Dis. 2020 Aug 25;14(8):e0008288. doi: 10.1371/journal.pntd.0008288 (PMC7473525; doi:10.1371/journal.pntd.0008288)
Supplement: S2 Text — (PDF) [file pntd.0008288.s007.pdf]

Tsetse population dynamics

We model changes in the numbers of adult tsetse and pupae over time and space on a lattice using two recursion equations. Movement of adult tsetse is assumed to be random and is approximated by a diffusion process, as previously described by Hargrove (2003).

Changes in the number of adults (A) at time t in cell i,j are described by:

$$A_{(i,j,t+1)} = (A_{(i,j,t)} + aC)(1 - \mu_B) + \beta P_{(i,j,t)} \tag{1}$$

where $C = A_{(i,j-1,t)} + A_{(i-1,j,t)} + A_{(i,j+1,t)} + A_{(i+1,j,t)} - 4A_{(i,j,t)}$ and $P_{(i,j,t)}$ is the number of pupae at time t in cell i,j. Each day, adults move to an adjacent cell with probability a and β represents the proportion of the pupal population emerging as adults on any given day. A proportion of adults (μ_B) then die due to natural, density-independent causes.

Changes in numbers of pupae are described by:

$$P_{(i,j,t+1)} = P_{(i,j,t)}(1 - \beta)(1 - \mu_P)(1 - P_{(i,j,t)}(1 - \beta P_{(i,j,t)})(1 - \mu_P)\delta) + lA_{(i,j,t)}/2 \tag{2}$$

where l is the proportion of females larvipositing each day and μ_P is the proportion of pupae which die each day due to density-independent processes. In addition pupal deaths result from density-dependent mortality, implemented using the coefficient δ.

We include additional adult tsetse mortality (μ_F) for tsetse in cells modelled as 'farming areas' where μ_F is a matrix with increased mortality (> 0) in cells $i, f_1 \geq j \leq f_2$ and zero otherwise so that Eq. 1 therefore becomes:

$$A_{(i,j,t+1)} = (A_{(i,j,t)} + aC)(1 - \mu_B)(1 - \mu_F) + \beta P_{(i,j,t)} \tag{3}$$

Tsetse population and trypanosome transmission dynamics

We extend the model in Eq. 2 and 3 to include trypanosome transmission. We use a single-vector, single-host system.

Our model assumptions generally follow those described by Rogers (1998) and Hargrove et al. (2012), except that we allow for a probability > 0 that non-teneral tsetse – flies that have taken at least one bloodmeal – can become infected with *T. brucei*, and for a decreased probability of infection with *T. congolense* for non-teneral flies, based on laboratory studies including Welburn and Maudlin (1992) and Kubi et al. (2006).

Each day, a proportion β_H of total hosts (N_H) in each cell produce offspring, which are susceptible (S_H). Susceptible hosts become exposed (E_H) with probability λ_H. Exposed hosts become infectious with probability σ_H and infected individuals (I_H) recover with probability φ. Recovered hosts (R_H) lose immunity and become susceptible with probability γ. All hosts die with probability μ_H.

Hosts:

$$S_{H(i,j,t+1)} = S_{H(i,j,t)}(1-\lambda_H)(1-\mu_H) + \gamma R_{H(i,j,t)}(1-\mu_H) + \beta_H N_{H(i,j,t)} \tag{4}$$

$$E_{H(i,j,t+1)} = E_{H(i,j,t)}(1-\sigma_H)(1-\mu_H) + \lambda_H S_{H(i,j,t)}(1-\mu_H) \tag{5}$$

$$I_{H(i,j,t+1)} = I_{H(i,j,t)}(1-\varphi)(1-\mu_H) + \sigma_H E_{H(i,j,t)}(1-\mu_H) \tag{6}$$

$$R_{H(i,j,t+1)} = R_{H(i,j,t)}(1-\gamma)(1-\mu_H) + \varphi I_{H(i,j,t)}(1-\mu_H) \tag{7}$$

where $\lambda_H = 1-(1-1/N_H)^{\alpha I_V p_H}$ where α the daily probability of tsetse biting and I_V the number of infectious tsetse.

Vectors:
In addition to tsetse reproduction, death and movement as described above, each day teneral (unfed) tsetse leave the susceptible teneral (S_V) class with probability α – the probability of taking a blood meal. A proportion λ_{VS} of these flies become exposed (E_{1V}), depending on the proportion of infected hosts and the probability p_S of becoming infected given an bite on an infected host, and the rest become susceptible non-teneral G_V. Susceptible non-teneral flies have probability p_G of becoming infected given a bite on an infected host. Infected flies move through three 'exposed' classes each with probability $3\sigma_V$ before developing a mature infection (I_V).

$$S_{V(i,j,t+1)} = (S_{V(i,j,t)} + aC_S)(1-\alpha)(1-\mu_B)(1-\mu_F) + \beta P_{(i,j,t)} \tag{8}$$

$$\begin{aligned} E_{1V(i,j,t+1)} = {} & (E_{1V(i,j,t)} + aC_{E1})(1-3\sigma_V)(1-\mu_B)(1-\mu_F) \\ & + \lambda_{VS}(S_{V(i,j,t)} + aC_S) + \lambda_{VG}(G_{V(i,j,t)} + aC_G) \end{aligned} \tag{9}$$

$$E_{2V(i,j,t+1)} = (E_{2V(i,j,t)} + aC_{E2})(1-3\sigma_V)(1-\mu_B)(1-\mu_F) + 3\sigma_V(E_{1V(i,j,t)} + aC_{E1}) \tag{10}$$

$$E_{3V(i,j,t+1)} = (E_{3V(i,j,t)} + aC_{E3})(1-\mu_B)(1-\mu_F)(1-3\sigma_V) + 3\sigma_V(E_{2V(i,j,t)} + aC_{E2}) \tag{11}$$

$$I_{V(i,j,t+1)} = (I_{V(i,j,t)} + aC_I)(1-\mu_B)(1-\mu_F) + 3\sigma_V(E_{3V(i,j,t)} + aC_{E3}) \tag{12}$$

$$G_{V(i,j,t+1)} = (G_{V(i,j,t)} + aC_G)(1-\lambda_{VG})(1-\mu_B)(1-\mu_F) + (1-\lambda_{VS})(S_{V(i,j,t)} + aC_S) \tag{13}$$

$$P_{(i,j,t+1)} = (P_{(i,j,t)} - \beta P_{(i,j,t)})(1-\mu_P)(1-(P_{(i,j,t)} - \beta P_{(i,j,t)})(1-\mu_P)\delta) + lN_{V(i,j,t)}/2 \tag{14}$$

.

where $\lambda_{VS} = \alpha p_S I_H/N_H$ and $\lambda_{VG} = \alpha p_G I_H/N_H$.

References

Welburn SC and Maudlin, I (1992). The nature of the teneral state in Glossina and its role in the acquisition of trypanosome infection in tsetse. *Ann. Trop. Med. Parasitol.*. 86, 529 – 536.

Rogers DJ (1998). A general model for the African trypanosomiases. *Parasitology.* 97,193 – 212.

Hargrove JW (2003). Optimized simulation of the control of tsetse flies Glossina pallidipes and G. m. morsitans (Dipteria: Glossinidae) using odour-baited targets in Zimbabwe. *Bull. Ent. Res.*. 93(1), 19 – 29.

Kubi C, Van Den Abbeele J, De Deken R, Marcotty T, Dorny P, van Den Bossche P. (2006). The effect of starvation on the susceptibility of teneral and non-teneral tsetse flies to trypanosome infection. *Med. Vet. Entomol.*. 20, 388 – 392.

Hargrove JW, Ouifki R, Kajunguri D, Vale GA and Torr SJ (2012). Modeling the control of trypanosomiasis using trypanocides or insecticide-treated livestock. *PLoS NTD.* Vol. 6(5),e1615.
