# Supplementary material for: Assessing the effect of insecticide-treated cattle on tsetse abundance and trypanosome transmission at the wildlife-livestock interface in Serengeti, Tanzania
Source: PLoS Negl Trop Dis. 2020 Aug 25;14(8):e0008288. doi: 10.1371/journal.pntd.0008288 (PMC7473525; doi:10.1371/journal.pntd.0008288)
Supplement: S3 Text — (DOCX) [file pntd.0008288.s008.docx]

**Model sensitivity analysis and model fitting**

**Sensitivity analysis for the tsetse population dynamics model**

We ran model simulations in R (R Core Team, 2014). For sensitivity analysis, rather than running simulations as described in Table 1 of the manuscript, using cells equivalent to the size of a square of 500 m and at 0.25-day time steps, to save computing time, we ran simulations in 0.5-day time steps, assuming each cell in the lattice is a square of side 1 km. The parameters and the ranges used for parameter values are described in Table S4.1.

**Table S4. 1. Parameters and values used in the model of tsetse population dynamics for sensitivity analysis.** Values used are per 0.5 days. For the diffusion coefficient (*a*) values are assuming each cell is a square of side 1 km.

| **Notation** | **Description** | **Range** | **Reference** |
| --- | --- | --- | --- |
| *l* | Probability female tsetse larviposits | 0.04 – 0.06 | (Denlinger & Ma, 1974; Hargrove, 2004) |
| *β* | Probability pupa emerges as an adult | 0.01 – 0.017 | (Hargrove, 2004; Phelps & Burrows, 1969) |
| *δ* | Pupal density-dependent mortality coefficient | 10^-5.3^ – 10^-4.35^ | NA |
| *µ_P_* | Pupal probability of mortality | 0.00125 – 0.005 | (Hargrove, 2004; Rogers & Randolph, 1984) |
| *µ_B_* | Adult baseline probability of mortality | 0.005 – 0.015 | (Hargrove, 2001) |
| *a* | Adult diffusion coefficient | 0.1 – 0.5 | (Hargrove, 1981) |
| *µ_F_* | Adult additional probability of mortality in farming areas | 0.015 – 0.25 | NA |

Assuming cells equivalent to a square of side 1 km, the total lattice comprised of 4 x 30 cells, with increased mortality in ‘farming areas’ between cells *j* = 21 and *j* = 30 (Figure S4.1). We sampled parameter space 1000 times using Latin hypercube sampling and ran the model for each resulting combination of parameter values. We produced scatter plots of tsetse density in cell *i* =1, *j* = 21 as a percentage of the tsetse density in cell *i* = 1, *j* = 1 (Figure S4.1) and using this model output, calculated the partial rank correlation coefficient (PRCC) for each parameter.

**Figure S4.1. Lattice used for sensitivity analysis of the tsetse population dynamics model.** Red cells highlight ‘farming’ area where increased mortality was applied. Orange cells indicate the area used for comparison of tsetse counts between wildlife and farming areas.

|  |  |  |  |  |  |  |  |  |  |  |  |  |  |  |  |  |  |  |  |  |  |  |  |  |  |  |  |  |  |
| --- | --- | --- | --- | --- | --- | --- | --- | --- | --- | --- | --- | --- | --- | --- | --- | --- | --- | --- | --- | --- | --- | --- | --- | --- | --- | --- | --- | --- | --- |
|  |  |  |  |  |  |  |  |  |  |  |  |  |  |  |  |  |  |  |  |  |  |  |  |  |  |  |  |  |  |
|  |  |  |  |  |  |  |  |  |  |  |  |  |  |  |  |  |  |  |  |  |  |  |  |  |  |  |  |  |  |
|  |  |  |  |  |  |  |  |  |  |  |  |  |  |  |  |  |  |  |  |  |  |  |  |  |  |  |  |  |  |

1,1 30,1

**Fitting the tsetse population dynamics model to the observed tsetse abundance data**

Model simulations, in 0.25 day time steps, were run on a lattice of 4 x 60 cells, each assumed to be of side 500 m, with increased mortality in ‘farming areas’ between cells *i_1:4_,j*_41:60,_ assuming for simplicity that flies in all cells in farming areas were subject to the same increased mortality.

For the observed tsetse catches, we rounded the distance from each tsetse trap to the wildlife boundary to the nearest 500 m before fitting the model to the data. Starting numbers of adults and pupae in the model were arbitrarily set to 5000/km^2^ and we ran the model for the equivalent of three years, in the presence of increased mortality in farming areas, to reach an equilibrium. We assumed that tsetse density scales linearly with numbers caught in traps and multiplied the observed tsetse counts by 100 to give an estimate of tsetse/ cell in the model, assuming traps catch c. 1 % tsetse in a 1 km^2^ area (Vale, Hargrove, Cockbill, & Phelps, 1986). We fitted the pupal density-dependent mortality coefficient (*δ*) as well as the additional adult mortality in farming areas (*µ_F_*) while holding other parameter values fixed. We used log_10_ transformed values of modelled and observed numbers of tsetse for fitting. For parameters with a PRCC >5 or < -5 from sensitivity analyses, we fitted the model using the maximum and minimum values for each to account for uncertainty in fixed parameter values (Table 1 of the manuscript).

**Sensitivity analysis for the trypanosome transmission model**

Due to uncertainty in parameter values for trypanosome transmission, to quantify the potential effect of the tsetse population decline on transmission across the interface, we first ran a sensitivity analysis without increased tsetse mortality. We carried out a sensitivity analysis of the model using Latin hypercube sampling of parameter space using the range of parameter values provided in Table 2 of the manuscript. Tsetse population dynamics parameters except *µ_V_* and *δ* were kept constant. The model was run for 1000 combinations of parameter values. We produced scatter plots of host and vector prevalence at equilibrium as a function of each of the trypanosome-related parameter values and calculated the PRCC for each parameter.

Denlinger, D. L., & Ma, W. C. (1974). Dynamics of the pregnancy cycle in the tsetse Glossina morsitans. *Journal of Insect Physiology*, *20*(6). https://doi.org/10.1016/0022-1910(74)90143-7

Hargrove, J. (1981). Tsetse dispersal reconsidered. *Journal of Animal Ecology*, *64*(2), 351–373. https://doi.org/10.1111/j.

Hargrove, J. (2001). Factors affecting density-independent survival of an island population of tsetse flies in Zimbabwe. *Entomologia Experimentalis et Applicata*, *100*(2), 151–164. https://doi.org/10.1023/A:1019271727810

Hargrove, J. (2004). Tsetse population dynamics. In I. Maudlin, P. Holmes, & M. Miles (Eds.), *The Trypanosomiases* (pp. 113–135). CABI Publishing.

Phelps, R., & Burrows, P. (1969). Prediction of the pupal duration of Glossina morsitans orientalis Vanderplank under field conditions. *Journal of Applied Ecology*, *6*(2), 323–337.

R Core Team. (2014). R: A language and environment for statistical computing. R Foundation for Statistical Computing.

Rogers, D. J., & Randolph, S. J. (1984). A review of density-dependent processes in tsetse populations. *Insect Science and Its Application*, *5*(5), 397–402.

Vale, G. a., Hargrove, J. W., Cockbill, G. F., & Phelps, R. J. (1986). Field trials of baits to control populations of Glossina morsitans morsitans Westwood and G. pallidipes Austen (Diptera: Glossinidae). *Bulletin of Entomological Research*, *76*, 179–193. https://doi.org/10.1017/S000748530001467X
