# Supplementary material for: Assessing the effect of insecticide-treated cattle on tsetse abundance and trypanosome transmission at the wildlife-livestock interface in Serengeti, Tanzania
Source: PLoS Negl Trop Dis. 2020 Aug 25;14(8):e0008288. doi: 10.1371/journal.pntd.0008288 (PMC7473525; doi:10.1371/journal.pntd.0008288)
Supplement: S1 Table — (DOCX) [file pntd.0008288.s009.docx]

**Fitted model parameter values.** Additional daily probability of adult tsetse mortality - *μ_F_,* pupal density-dependent mortality coefficient – *δ.* Values in parentheses are standard errors.

| **Fixed *a* (km/day)** | **Fitted *μ_B_*** | **Fitted value *δ*** |
| --- | --- | --- |
| **Larviposition 0.125/day** |  |  |
| 0.25 | 0.097 (0.0101) | 1.25 x 10^-5^ (1.77 x 10^-6^) |
| 0.5 | 0.164 (0.0185) | 1.01 x 10^-5^ (1.45 x 10^-6^) |
| 1 | 0.289 (0.0334) | 7.91 x 10^-6^ (1.42 x 10^-6^) |
| **Larviposition 0.1/day** |  |  |
| 0.25 | 0.086 (0.0097) | 6.97 x 10^-6^ (8.71 x 10^-7^) |
| 0.5 | 0.152 (0.0181) | 4.78 x 10^-6^ (6.86 x 10^-7^) |
| 1 | 0.276 (0.0329) | 3.63 x 10^-6^ (5.34 x 10^-7^) |
| **Larviposition 0.08/day** |  |  |
| 0.25 | 0.077 (0.0092) | 2.28 x 10^-6^ (3.67 x 10^-7^) |
| 0.5 | 0.142 (0.0175) | 1.67 x 10^-6^ (2.82 x 10^-7^) |
| 1 | 0.264 (0.0323) | 1.00 x 10^-6^ (1.87 x 10^-7^) |
